# Serine Protease EspP from Enterohemorrhagic *Escherichia Coli* Is Sufficient to Induce Shiga Toxin Macropinocytosis in Intestinal Epithelium

**DOI:** 10.1371/journal.pone.0069196

**Published:** 2013-07-18

**Authors:** Julie In, Valeriy Lukyanenko, Jennifer Foulke-Abel, Ann L. Hubbard, Michael Delannoy, Anne-Marie Hansen, James B. Kaper, Nadia Boisen, James P. Nataro, Chengru Zhu, Edgar C. Boedeker, Jorge A. Girón, Olga Kovbasnjuk

**Affiliations:** 1 Department of Medicine, Johns Hopkins University School of Medicine, Baltimore, Maryland, United States of America; 2 Department of Cell Biology, Johns Hopkins University School of Medicine, Baltimore, Maryland, United States of America; 3 Department of Microbiology & Immunology, University of Maryland School of Medicine, Baltimore, Maryland, United States of America; 4 Department of Pediatrics, University of Virginia School of Medicine, Charlottesville, Virginia, United States of America; 5 Department of Medicine, University of New Mexico School of Medicine, Albuquerque, New Mexico, United States of America; 6 Department of Molecular Genetics and Microbiology, Emerging Pathogens Institute, University of Florida, Gainesville, Florida, United States of America; University of Osnabrueck, Germany

## Abstract

Life-threatening intestinal and systemic effects of the Shiga toxins produced by enterohemorrhagic *Escherichia coli* (EHEC) require toxin uptake and transcytosis across intestinal epithelial cells. We have recently demonstrated that EHEC infection of intestinal epithelial cells stimulates toxin macropinocytosis, an actin-dependent endocytic pathway. Host actin rearrangement necessary for EHEC attachment to enterocytes is mediated by the type 3 secretion system which functions as a molecular syringe to translocate bacterial effector proteins directly into host cells. Actin-dependent EHEC attachment also requires the outer membrane protein intimin, a major EHEC adhesin. Here, we investigate the role of type 3 secretion in actin turnover occurring during toxin macropinocytosis. Toxin macropinocytosis is independent of EHEC type 3 secretion and intimin attachment. EHEC soluble factors are sufficient to stimulate macropinocytosis and deliver toxin into enterocytes *in vitro* and *in vivo*; intact bacteria are not required. Intimin-negative enteroaggregative *Escherichia coli* (EAEC) O104:H4 robustly stimulate Shiga toxin macropinocytosis into intestinal epithelial cells. The apical macropinosomes formed in intestinal epithelial cells move through the cells and release their cargo at these cells’ basolateral sides. Further analysis of EHEC secreted proteins shows that a serine protease EspP alone is able to stimulate host actin remodeling and toxin macropinocytosis. The observation that soluble factors, possibly serine proteases including EspP, from each of two genetically distinct toxin-producing strains, can stimulate Shiga toxin macropinocytosis and transcellular transcytosis alters current ideas concerning mechanisms whereby Shiga toxin interacts with human enterocytes. Mechanisms important for this macropinocytic pathway could suggest new potential therapeutic targets for Shiga toxin-induced disease.

## Introduction

Shiga toxin (Stx)-producing bacteria (STEC) are major foodborne pathogens. No current therapy specifically prevents the broad spectrum of devastating STEC intestinal and systemic diseases that include hemorrhagic colitis, hemolytic uremic syndrome (HUS) and seizures [1–4]. The two major immunologically distinct toxin forms, Stx1 and Stx2, share almost 60% sequence identity but vary in potency [5,6]. Stx2 is more strongly associated with severe human disease. Past STEC outbreaks have been linked predominantly to enterohemorrhagic *E. coli* (EHEC), especially the O157:H7 strain. EHEC strains produce characteristic attaching and effacing (A/E) lesions on enterocytes [7]. These lesions have been attributed to products of the locus of enterocyte effacement (LEE) pathogenicity island. The LEE includes the type 3 secretion system (T3SS), T3SS effectors and the *eae* island that encodes the major EHEC adhesin, intimin [6–9]. It has been suggested that the combination of Stx and intimin expression is required for full virulence [10,11]. However, a recent STEC outbreak caused by the intimin-negative O104:H4 EAEC strain appears to show that Stx is the major virulence factor [12,13] and intimin adhesion can be replaced by other adherence factors.

All toxin-induced complications start from the interactions between gut luminal Stx and intestinal epithelial cells (IEC), especially abundant enterocytes. Earlier hypotheses concerning mechanisms of Stx action on enterocytes were dominated by ideas that glycosphingolipids Gb3 and/or Gb4 serve as Stx receptors [14–16]. Gb3-mediated retrograde toxin trafficking was postulated to be key for EHEC-induced enterocyte damage. By contrast, more recent data [17–21] shows that human enterocytes bind neither Stx1 nor Stx2 either normally or during EHEC infection due to the lack of Gb3 receptors. Gb4 serves as a receptor for only the nonpathogenic Stx2e isoform in humans [22]. These results have required rethinking of the previous models for EHEC intestinal disease.

Upon STEC infection, both small intestinal and colonic enterocytes are intoxicated with Stx1 and Stx2 by Gb3 receptor-independent uptake mechanisms [21]. We have shown that EHEC infection increases Stx1 and Stx2 uptake in IEC by stimulation of macropinocytosis (MPC) [20]. MPC provides an efficient route for uptake of macromolecules by an actin-driven but receptor-, clathrin- and caveolin-independent mechanism [23,24]. Stx is found inside F-actin-coated macropinosomes which traffic from the apical to basolateral side of IEC [20]. Toxin MPC increases transcellular transcytosis [20], which may facilitate systemic toxin spread and subsequent damage to kidneys and the central nervous system. EHEC-stimulation of macropinocytic blebs depends on Cdc42 and the non-muscle myosin II A (NMIIA). Modulating Cdc42 and NMIIA in EHEC-infected cells by either pharmacologic or molecular approaches significantly influences Stx uptake [20]. However, the bacterial factors necessary for actin rearrangement upon MPC stimulation remain uncharacterized.

The A/E lesions characteristic of EHEC infection include actin pedestals beneath the intimately attached bacteria at the apical surface of IEC. It is well established that actin rearrangement necessary for pedestal formation requires active type 3 secretion and intimin [25–27]. We now report an investigation of the roles of T3SS and intimin in toxin MPC *in vitro* and *in vivo*. We tested the hypothesis that stimulation of MPC is a by-product of the host actin rearrangement involved in EHEC pedestal formation.

## Results

### Functional T3SS and expression of full length intimin are not necessary for EHEC-stimulated Stx1 and Stx2 macropinocytosis in IEC

To test the hypothesis that T3SS-induced actin remodeling is necessary for MPC stimulation, we treated T84 cells for 4h at 37°C with **a)** 0.3 µg/mL Stx1-680 (control conditions) or toxin plus toxin-negative EHEC strain **b)** EDL933, **c)** O157:H7, **d)** EDL933 T3SS deletion mutant of *E. coli* Secreted Protein A (Δ*espA*), which forms a filament that serves to translocate T3SS effectors from the bacterium into the host cell, **e)** O157:H7 truncation mutant [28] of a major EHEC adhesin intimin (Δ*eae*), which is necessary for the attachment of the bacterium to the host cell, or **f)** the non-pathogenic laboratory strain *E. coli* K-12. Both EDL933 and O157:H7 significantly increased Stx1 uptake by T84 cells, while, as expected, K-12 did not (Table 1). Both mutants Δ*espA* and Δ*eae* stimulated toxin uptake in T84 cells similar to the corresponding wild type strains, demonstrating that EHEC-induced actin remodeling necessary for Stx1 MPC does not require active EspA-dependent type 3 secretion or expression of functional intimin.

**Table 1:** Secretion by T3SS or expression of full length intimin are not involved in EHEC-stimulated MPC of Stx.

**Experimental conditions**	**Stx1/GAPDH uptake**			
	**Mean (%)**	**SEM**	**n**	**p value**
No bacteria (control)	100		59	
EDL933	486*	79	36	0.006
EDL933 Δ*espA*	466*	65	7	0.03
O157:H7	526*	35	7	0.01
O157:H7 Δ*eae*	531*	35	7	0.04
*E. coli* K-12	96	15	3	NS

Stx1 uptake in T84 cells is stimulated by either EHEC wild type or EHEC Δ*espA* or EHEC Δ*eae* deletion mutants but not by non-pathogenic *E. coli* K-12; * - significant vs. control; NS - not significant compared to control; n number of monolayers.

### EHEC soluble factors are sufficient to stimulate toxin MPC in IEC *in vitro*

**Figure 1:**
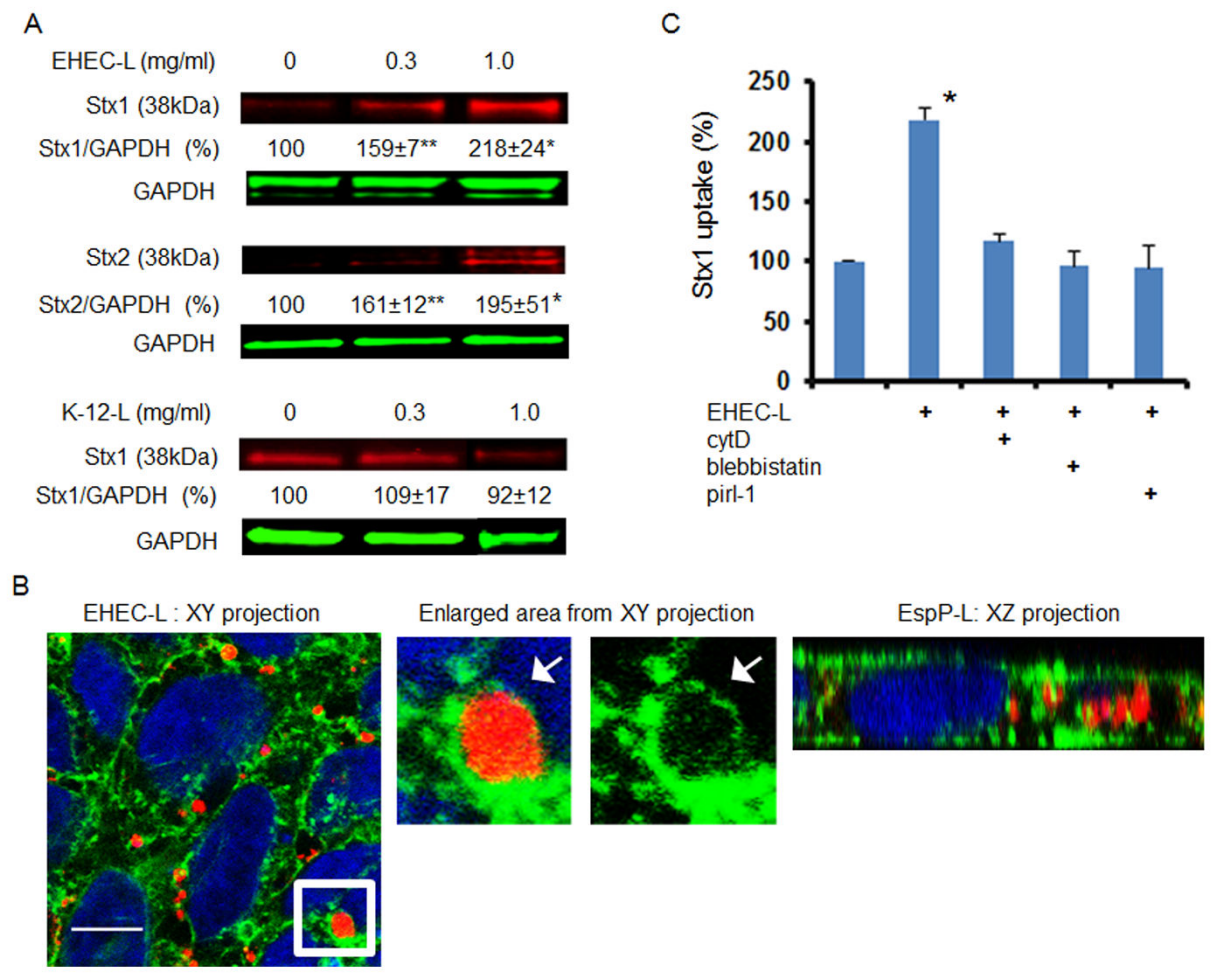
EHEC-L stimulates Stx1 and Stx2 uptake in T84 cells, while lysate from *E. coli* K12 strain does not. (A) Representative immunoblots (IB) and quantitative representations of IB data show that increasing concentrations of EHEC-L significantly increase Stx1 and Stx2 uptake in T84 cells compared to untreated cells and cells treated with increasing concentrations of K12-L (n ≥ 6 monolayers per each experimental condition from 3 independent experiments; * - significant compared to the control (p < 0.05); ** - significant compared to control (p < 0.001). (B) Representative XY and XZ confocal optical section through T84 cells incubated for 4 h with EHEC-L (1 mg/mL) and Stx2 (0.5 µg/mL) show that EHEC reorganized actin in T84 cells and triggered the formation of actin coated macropinosomes of different sizes and many of the F-actin vesicle carry Stx2. Enlarged area from XY section (white box) shows that Stx1B is contained inside the F-actin-coated macropinosome. F-actin - green by phalloidin-AlexaFluor 488; Stx2 - red by AlexaFluor 568; nuclei – blue by Hoechst, bar -10 µm. (C) EHEC-L-induced Stx1 uptake was reduced to the control level in the presence of inhibitors of MPC including cytD, blebbistatin or pirl-1 (* - significant compared to control, p = 0.012; n ≥ 6 monolayers per each experimental condition from 3 independent experiments).

To further show that the process of actin rearrangement necessary for EHEC pedestal formation is different from that involved in MPC, we treated T84 cells with EHEC lysate (EHEC-L). The T84 cells treated with lysates prepared from strain EDL933 significantly increased Stx1 and Stx2 uptake in a lysate concentration-dependent manner relative to control T84 cells treated with toxin only or with lysate from *E. coli* K-12 strain (K-12-L; Figure 1A). Thus, EHEC-L duplicates the effect of intact EHEC on stimulation of Stx1 and Stx2 uptake by IEC.

Next we examined the EHEC-L-induced changes in F-actin and toxin distribution using confocal microscopy. T84 cells were incubated for 4 h with 1 mg/mL EHEC-L in the presence of Stx2-568, and then washed, fixed, and immunostained to detect F-actin and nuclei. In contrast to untreated control (Figure S1), EHEC-L rearranged the F-actin fibers into macropinosomes all through the cell volume (Figure 1B) and particularly near the lateral membranes, the sites of F-actin enrichment. Significant numbers of macropinosomes were filled with the toxin. The appearance of EHEC-L-induced changes in F-actin and the distribution of Stx2 were similar to those reported in T84 cells infected with the EDL933 strain [20]. The number of apical macropinocytic blebs (≥ 2 µm) in EHEC-L-treated cells was 16.3 ± 1.9 blebs /100 cells (778 cells analyzed from 6 independent preparations), which was significantly higher (p < 0.00001) compared to 2 ± 0.3 blebs /100 cells (420 cells were analyzed from 3 independent preparations) in control cells, in which macropinocytic blebs occur due to a basal unstimulated MPC [29]. Among 778 analyzed EHEC-L treated cells, 385 cells (~49%) contained Stx2-positive macropinosomes.

We further hypothesized that if EHEC-L-stimulated toxin uptake is mediated by MPC, the molecular mechanism of MPC should resemble induction by intact bacteria [20], a process that requires actin remodeling executed by activation of the small GTPase Cdc42 and motor protein NMIIA.

**Figure 2:**
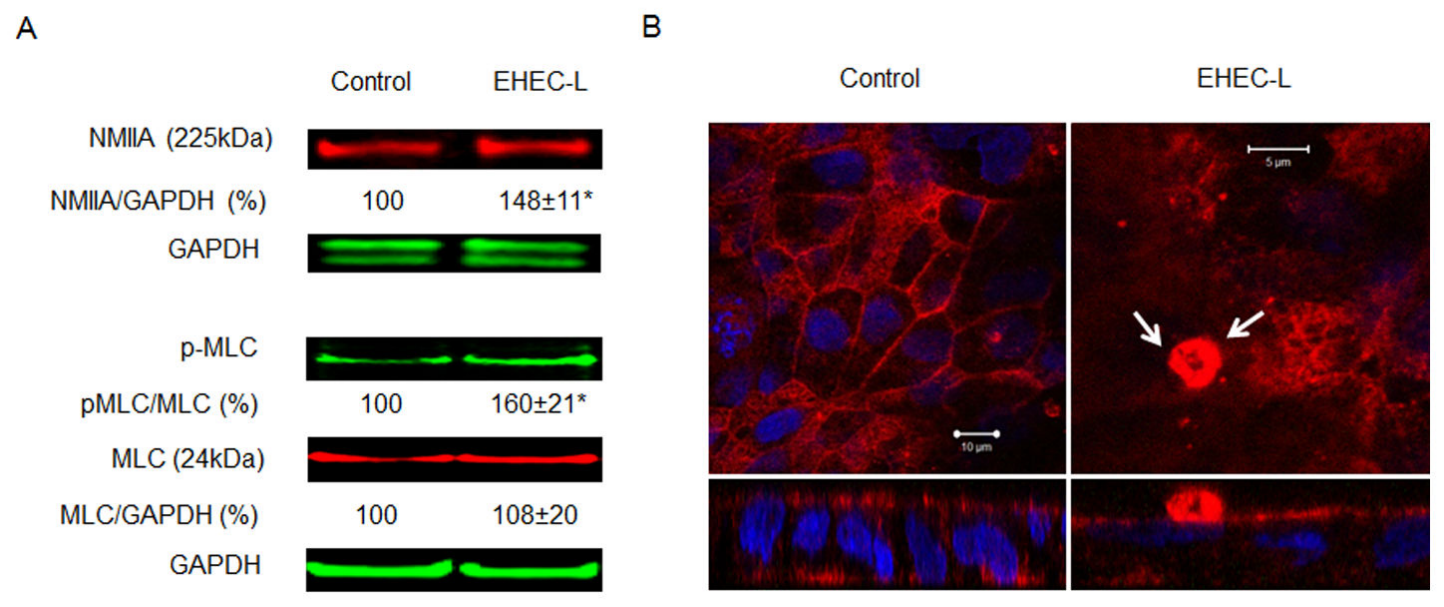
NMIIA and MLC are involved in EHEC-L-induced Stx MPC. (A) Representative IB and quantitative data show that EHEC-L-induced MPC is accompanied by a significant increase in the relative amount of NMIIA and increase in pMLC (* - significant compared to the control (p < 0.01); n ≥ 4 monolayers per each experimental condition from 3 independent experiments. (B) Representative XY confocal optical sections through the apical region of T84 cells and corresponding XZ projections show the difference in MLC (red) distribution between control and EHEC-L-treated monolayers. White arrows indicate that upon EHEC-L treatment the MLC is concentrated in an apical macropinocytic bleb. In both panels: MLC-red by AlexaFluor568; nuclei – blue by Hoechst. Analysis of 23 apical F-actin blebs in EHEC-L treated cells from 2 independent experiments show that all 23 counted apical blebs were MLC-positive.

EHEC-L-stimulated Stx1 uptake is actin-dependent and completely inhibited by cytochalasin D (cytD), a cell-permeable inhibitor of actin polymerization (Figure 1C). The EHEC-L-induced actin remodeling was accompanied by NMIIA upregulation (Figure 2A), and blebbistatin, a specific pharmacological inhibitor of NMII ATPase activity [30,31], completely inhibited Stx1 uptake (Figure 1C). NMIIA activity also requires phosphorylation of myosin regulatory light chain (MLC) [32]. Incubation of T84 cells with EHEC-L significantly increased the MLC phosphorylation (pMLC; Figure 2A). Moreover, in EHEC-L treated T84 cells, MLC was redistributed from the brush border (BB) membrane and perijunctional ring into the macropinocytic blebs (Figure 2B). Treatment of T84 cells with the K-12-L changed neither the pMLC nor the MLC distribution compared to control cells (data not shown). EHEC-L-induced MPC is also a Cdc42 dependent process and pirl-1, a specific Cdc42 inhibitor [33,34], significantly decreased toxin uptake in EHEC-L-treated IEC (Figure 1C). We conclude that EHEC soluble factors, but not intact bacteria, are sufficient to stimulate toxin MPC in IEC.

### The actin remodeling required for MPC differs from that necessary for EHEC pedestal formation

It has been previously suggested that EHEC infection may trigger multiple pathways of actin assembly in host cells [35,36]. Data presented here indicate that actin remodeling necessary for MPC differs from that involved in EHEC intimate attachment to the enterocytes, which is a T3SS-dependent process. It has been shown that EHEC controls attachment to the host cells through a tightly regulated balance between tyrosine phosphorylation and dephosphorylation of cortactin, the F-actin binding protein which is involved in pedestal formation [36–38]. This occurs through direct binding between phosphorylated cortactin (p-cortactin) and the T3SS effectors Tir and EspFu. P-cortactin thus serves to link these two EHEC effectors to the actin polymerization machinery of the host cells. These interactions cause dephosphorylation of multiple tyrosine residues on human cortactin including Y470 (p-cortactinY470) and redistribution of cortactin throughout the entire pedestal. Thus, a functional T3SS is necessary for EHEC-induced cortactin dephosphorylation and actin pedestal formation.

**Figure 3:**
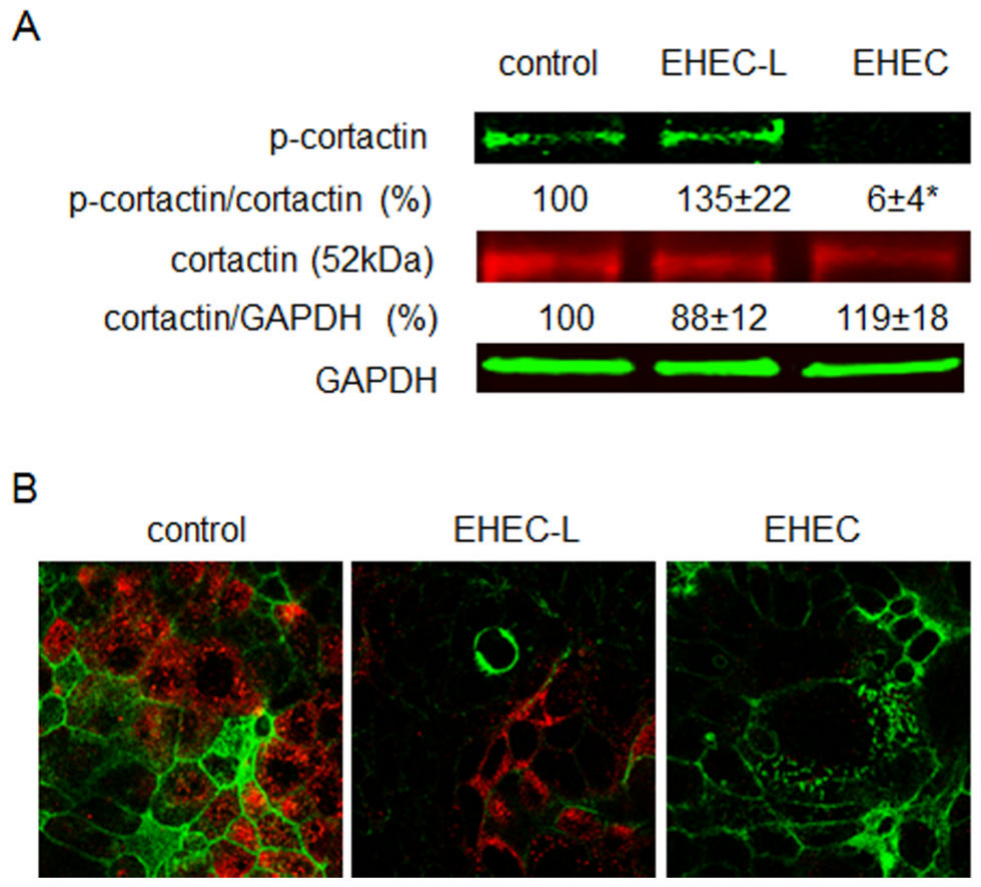
**Cortactin is not involved in EHEC-L-induced MPC.** (A) Representative IB and quantitative data show that treatment of T84 cells with EHEC-L does not affect the phosphorylation of cortactin (p-cortactin) in contrast to EHEC infection, which almost completely dephosphorylates cortactin (* p = 0.0006); n = 6 monolayers from 3 independent experiments). (B) Representative XY confocal optical sections through the apical region of T84 cells show that p-cortactin (red) is absent from the apical macropinocytic blebs detected by F-actin (green), but is present in surrounding cells not involved in MPC similar to that in control conditions. Analysis of 27 apical macropinocytic blebs in EHEC-L treated cells from 3 independent preparations showed no presence of p-cortactin in F-actin blebs. Also, p-cortactin is virtually absent from EHEC infected T84 monolayers. In both panels: p-cortactin – red by AlexaFluor568; F-actin – green by phalloidin-AlexaFluor488; nuclei – blue by Hoechst.

In agreement with these published observations, infection of T84 cells with EDL933 (Figure 3A) or O157:H7 (data not shown) strains significantly decreased p-cortactinY470, while EHEC-L treatment did not change the p-cortactinY470 compared to control untreated T84 cells (Figure 3A). Moreover, p-cortactin was absent from EHEC-L-induced F-actin macropinocytic blebs (Figure 3B). These data further show that actin remodeling necessary for MPC is independent of T3SS activity and differs from actin rearrangement associated with the formation of F-actin pedestals.

### Activation of the non-receptor tyrosine kinase Src by EHEC infection is not necessary for stimulation of MPC

**Figure 4:**
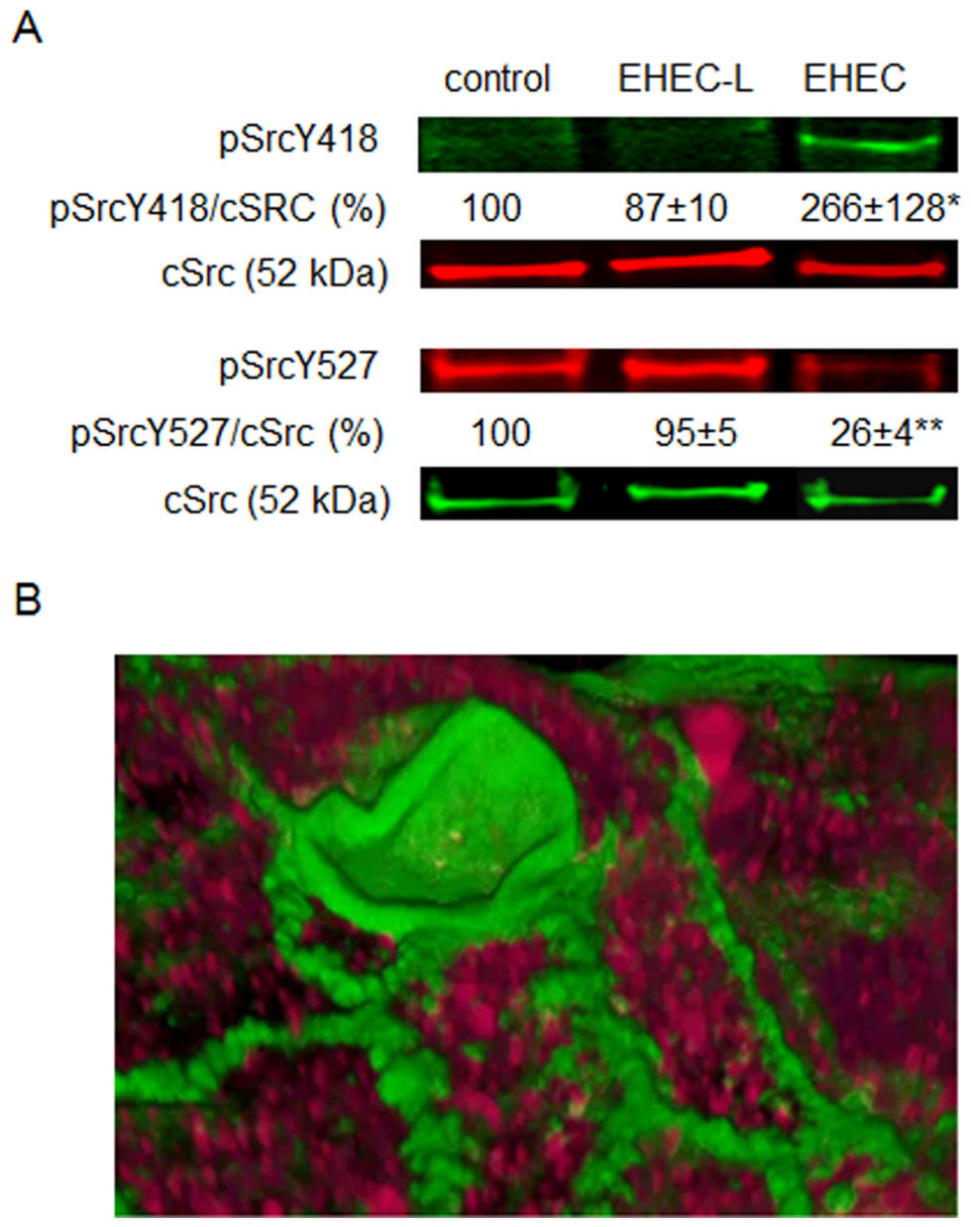
**Src activation by EHEC infection is not involved in EHEC-stimulated MPC.** (A) Representative IB and quantitative data show that treatment of T84 cells with EHEC-L does not activate Src (pSrcY418) in contrast to EHEC infection, which significantly increases the relative amount of pSrcY418 (* p = 0.044); n = 20 monolayers from10 independent experiments) and significantly decreases the relative amount of inactive pSrcY527 (** p = 0.0001, n = 20 monolayers from 10 independent experiments), while the relative amount of cSrc remains constant. (B) Representative 3D reconstruction of confocal optical sections through the apical region of T84 cells infected with EHEC strain EDL933 show that active pSrcY418 (red) is absent from the macropinocytic blebs detected by F-actin (green), but is present all though the cells. Analysis of 18 apical macropinocytic blebs in EDL933-infected cells from 2 independent preparations showed no presence of pSrcY418 in F-actin blebs. In panel B: pSrcY418 – red by AlexaFluor568; F-actin - green by phalloidin-AlexaFluor488.

Activation of non-receptor tyrosine kinase Src is often considered a necessary step in the initiation of host signaling leading to stimulation of macropinocytic actin remodeling in epithelial cells [21,24]. Thus, we tested the role of Src activation in toxin MPC. While treatment of T84 cells with intact EHEC significantly increases the relative amount of active phosphorylated Src (pSrc418) and significantly decreases the relative amount of inactive pSrc529, EHEC-L does not activate Src (Figure 4A). Moreover, active pSrc418 was excluded from F-actin macropinocytic blebs induced by intact EHEC (Figure 4B), indicating that EHEC-induced Src activation is not involved in EHEC-stimulated MPC.

### EHEC-L is sufficient to stimulate toxin MPC in IEC *in vivo*

**Figure 5:**
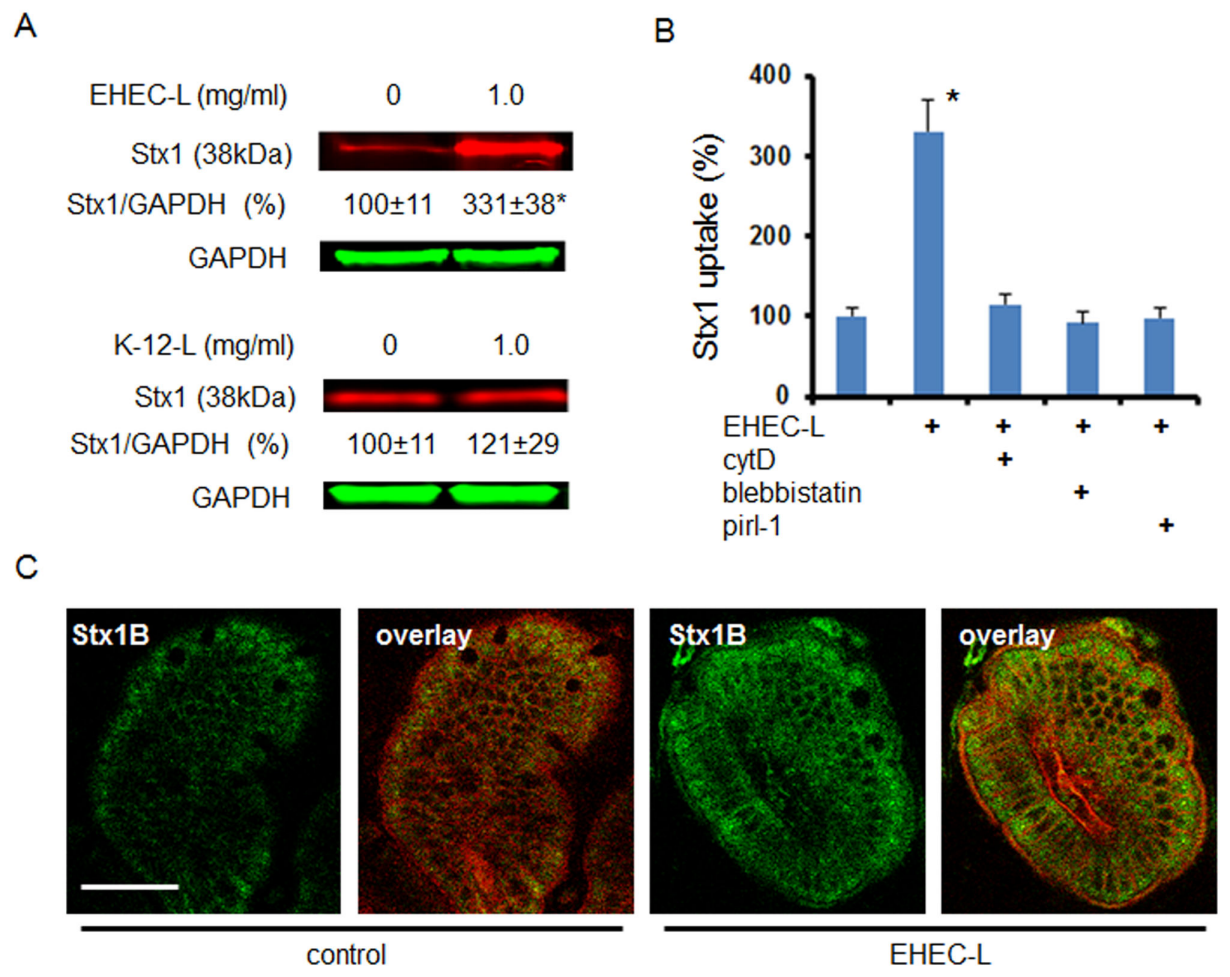
**EHEC-L stimulate Stx1 MPC in mouse ileum.** (A) Representative IB and quantitative representations of data show that EHEC-L significantly increases Stx1 uptake in mouse enterocytes compared to tissue treated with K-12-L (n ≥ 6 animals per each experimental condition; * - significant compared to the control (p = 0.03)). (B) EHEC-L-induced Stx1 uptake in mouse intestine was reduced to the control level in the presence of inhibitors of MPC including cytD (n = 3 mice), blebbistatin (n = 4 mice) or pirl-1 (n = 4 mice). (C) Representative multiphoton optical section either through control sample of ileal tissue exposed to Stx1 only or tissue treated with EHEC-L plus Stx1 shows substantial increase in Stx1 fluorescence inside the enterocytes. In both panels: plasma membranes – red by tdTomato, Stx1-488 – green; bar -50 µm.

The major issue with murine models of EHEC infection is insufficient intestinal colonization by human EHEC strains [6,39]. Our *in vitro* observation that EHEC-induced MPC is independent of bacterial attachment suggests that EHEC-L also might stimulate Stx uptake in mouse intestine. To test this hypothesis we adopted a previously described mouse intestinal loop model [40,41]. Exposure of mouse small intestinal loop for 4 hours to the mixture of Stx1 and EHEC-L significantly increased Stx1 uptake by IEC compared to the toxin alone or to the mixture of Stx1 and K-12-L (Figure 5A).

Next we compared the molecular mechanism of EHEC-L-stimulated toxin uptake in mouse intestine to this process in T84 cells. EHEC-L-stimulated toxin uptake *in vivo* is actin-dependent and is significantly inhibited by cytD and blebbistatin (Figure 5B). Actin remodeling necessary for EHEC-L-stimulated toxin uptake *in vivo* is also Cdc42 dependent, as pirl-1 significantly decreases toxin uptake in EHEC-L-treated mouse enterocytes (Figure 5B).

Taken together, these data show that EHEC-L-stimulated MPC in IEC *in vivo* and *in vitro* is similar to that stimulated by intact EHEC. These data further demonstrate that EHEC-stimulated toxin uptake by mouse enterocytes is a T3SS-independent process.

Additionally, we visualized the distribution of non-catalytic B-subunit of Stx1 (Stx1B) in the mouse small intestine (Figure 5C) and quantified the relative toxin amount inside the cells in the presence or absence of EHEC-L. The average fluorescence intensity of intracellular Stx1B significantly increased (p = 0.00196) from 1319 ± 32 grey levels (g. l.) in control tissue to 1637 ± 26 g. l. in tissue treated with EHEC-L (n = 40 optical sections from 2 intestinal preparations per experimental condition).

EHEC-L also significantly (p = 0.038) increased the uptake of 0.5 mg/mL 70 kDa dextran-AlexaFluor 488 by mouse IEC, determined from the analysis of confocal images by quantification of average fluorescence intensity of intracellular dextran in control tissue (274 ± 123.1 g. l.) and in tissue treated with EHEC-L (617.3 ± 87.4 g. l. ; n= 40 optical sections from 2 intestinal preparations per experimental condition). We conclude that EHEC soluble factors present in EHEC-L are sufficient to stimulate the uptake of Stx1 and other high molecular weight cargo in mouse enterocytes *in vivo*.

### Lysate from intimin-negative strain of Enteroaggregative *E. coli* (EAEC) also stimulates toxin endocytosis

**Figure 6:**
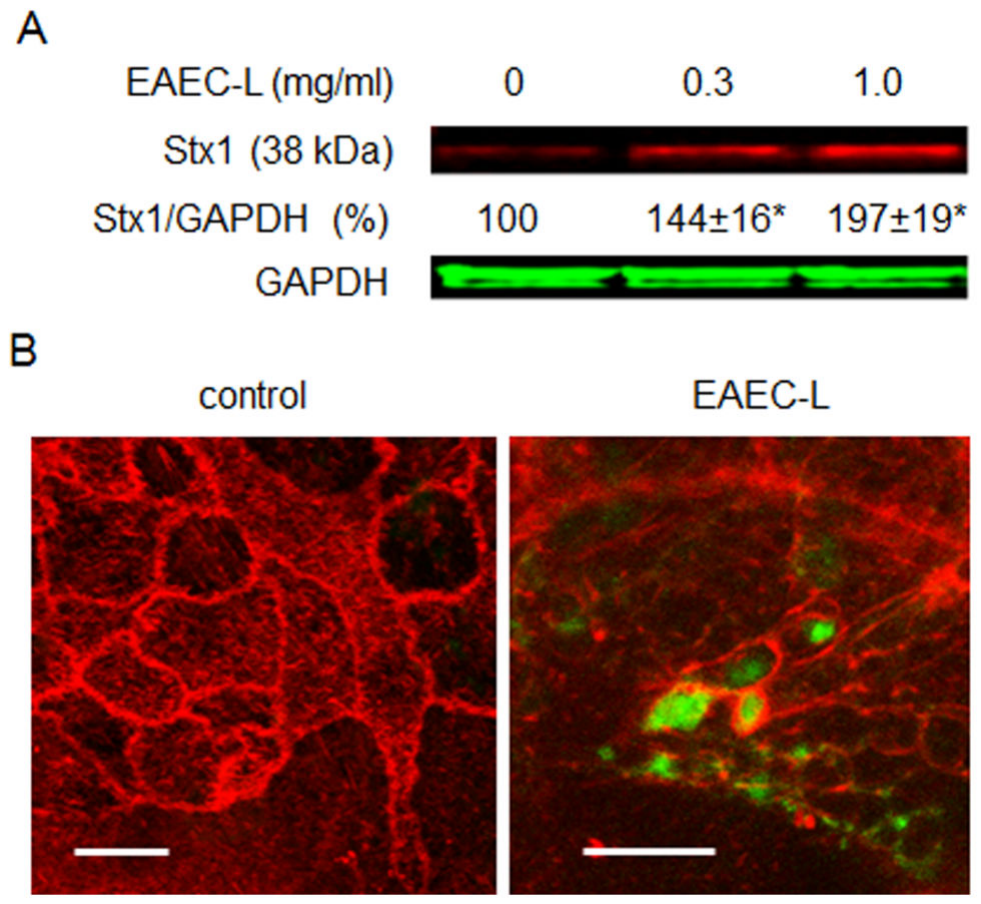
**EAEC-L stimulates Stx1 uptake in T84 cells by stimulation of MPC.** (A) Representative IB and quantitative representations of IB data show that increasing concentrations of EAEC-L significantly increased Stx1 uptake in T84 cells compared to untreated cells (n ≥ 3 monolayers per each experimental condition; * - significant compared to the control (p < 0.05)). (B) Representative XY optical sections through either control or EAEC-L-treated T84 cells additionally incubated with Stx1B-488 for 4 h show EHEC-L induced actin remodeling with formation of F-actin coated macropinosomes (spherical or irregularly shaped). Numerous macropinosomes carry the Stx1B-488 (green). F-actin - red by phalloidin -Alexa Fluor 568; bars -5 µm.

Recent severe STEC outbreak in Europe has been linked to an intimin-negative EAEC H104:O4 strain producing Stx2 [12,13]. However, the manifestation of disease caused by this Stx2-producing EAEC was very similar to that caused by EHEC infection. Using transmission electron microscopy (TEM) it has been shown that EAEC (H104:O4) colonization of T84 cells caused blebbing of the apical membrane and cell “vacuolarization” [42] which closely resemble the macropinosomes in EHEC-infected T84 cells [20]. These data together with our finding that expression of full length intimin by EHEC is not necessary for MPC suggests that intimin-negative Stx-producing *E. coli* other than EHEC might also use MPC as a way to deliver the toxin into the enterocytes. To test this hypothesis we examined the effect of a lysate prepared from EAEC H104:O4 strain (EAEC-L) on Stx1 uptake. EAEC-L significantly increases toxin endocytosis in T84 cells in a lysate concentration-dependent manner (Figure 6A). Examination of T84 cells treated with EAEC-L and Stx1B revealed the F-actin nature of the apical blebs as well as the F-actin coated macropinosomes inside the cells (Figure 6B). Many macropinosomes carried Stx1B similar to that detected in T84 cells treated with EHEC-L (Figure 1B) or infected with EHEC [20]. These data suggest that similar to EHEC, the EAEC O104:H4 strain expresses soluble factors sufficient to trigger actin remodeling necessary for MPC and that live STEC bacteria are not necessary for stimulation of MPC.

### EHEC-L-stimulated MPC transports cargo across the IEC

To cause HUS and other systemic complications, Stx must be transported from the intestinal lumen across the epithelial layer to the serosal side. We have previously shown that upon EHEC infection of T84 cells, the toxin trapped inside the actin-coated macropinosomes was transferred across the cells and was released at the basolateral side into the medium. Thus, EHEC-stimulated MPC caused significant increase in toxin transcellular transcytosis compared to T84 cells exposed to toxin only [20]. Consequently, we tested whether MPC caused by EHEC-L is sufficient to stimulate the transcytosis of macropinocytic cargo. First we examined the formation and intracellular distribution of macropinosomes using TEM. For these experiments, control T84 cells or cells treated with either EHEC-L or intact EHEC bacteria were incubated apically with horseradish peroxidase (HRP), a classical marker of MPC which is also readily detectable by TEM [20,24,43].

**Figure 7:**
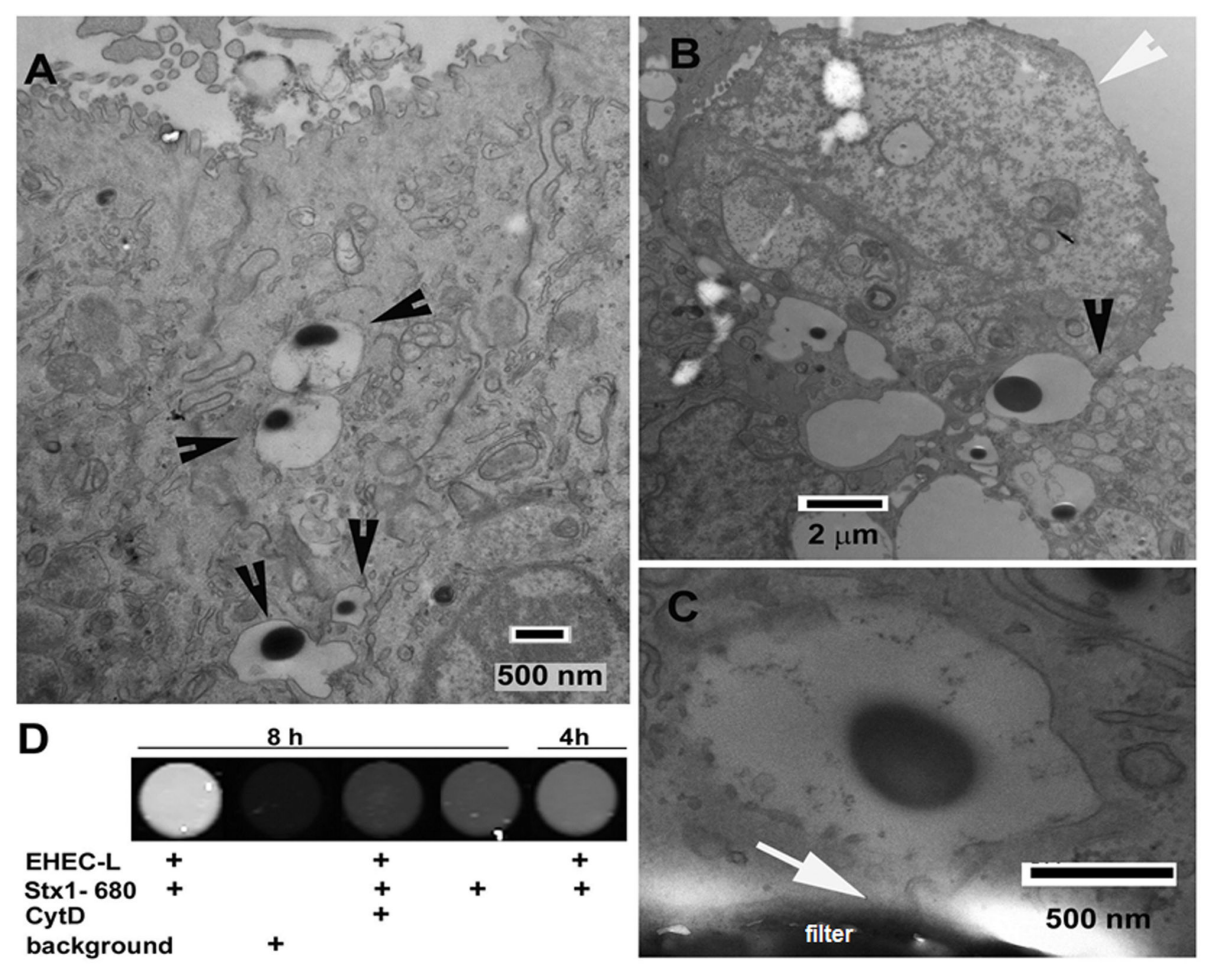
**EHEC-L induced MPC leads to the transcellular transcytosis of the apical cargo.** (A) Representative TEM image of T84 cells treated apically for 4h with a mixture of EHEC-L and 1 mg/mL HRP. EHEC-L causes the formation of macropinosomes filled with HRP (black arrowheads). (B) Representative TEM image depictures the process of a formation of HRP-bearing macropinosomes (black arrowhead). The apical EHEC-L induced bleb (white arrowhead) upon retraction back into the cell [19] and closure forms a new HRP-containing macropinosome. (C) Representative TEM image shows that the HRP-bearing macropinosome is reaching the basolateral side of filter-grown T84 cells (white arrow) and makes contact with the basal membrane. (D) Representative image obtained from fluorescence plate reader shows that EHEC-L stimulates Stx1 transcytosis in a time-dependent manner. This transcytosis is significantly inhibited by cytD (Table 2).

Both the EHEC-L and the bacteria damage the microvilli and cause the massive appearance of macropinosomes, often of large size and irregular shapes (Figure 7A, S2 and S3). The presence of HRP in the majority of these macropinosomes indicates that macropinosomes emanate from the apical surface of IEC upon macropinocytic bleb retraction (Figure 7B) and are involved in HRP endocytosis. Quantification of the total number of macropinosomes per cell upon EHEC-L treatment varied between 2 and 30 per image and was similar in EHEC-infected cells (n=9 images of equal magnification per condition, 2 independent experiments). However, in monolayers infected with EHEC the percent of macropinosome-containing cells was 65 ± 9%, which was significantly higher (p = 0.0013) compared to that in EHEC-L-exposed monolayers (32 ± 5%). These data suggest that both EHEC-L and intact EHEC stimulate MPC by a similar mechanism with similar outcome. However, EHEC infection might constantly supply a higher concentration of “active ingredients” necessary for MPC stimulation and by this may achieve a higher efficiency of MPC compared to the EHEC lysates.

Importantly, HRP-positive macropinosomes were detected not only apically and sub-apically (Figure 7A-B), but also basolaterally, with some of them making contact with the basal membrane (Figure 7C), indicating that macropinosomes might be involved in directional apical-to-basal trafficking and delivery of high molecular weight cargo (e.g. Stx or HRP or other bacterial products) from the apical to the basolateral side of the intestinal epithelial monolayer [20]. These observations further suggest that EHEC-L-stimulated MPC might also cause an increase in transcellular transcytosis of the cargo, which we addressed next.

Treatment of T84 cells with EHEC-L significantly increased Stx1 transcytosis in a time-dependent manner (Figure 7D and Table 2) similar to the effect of intact EHEC [20]. Toxin transcytosis was actin-dependent and almost completely inhibited by treatment of cells with cytD (Figure 7D and Table 2). The effect of EHEC-L on transcytosis was not Stx specific and transcytosis of HRP and 40 kDa dextran, both labeled with Alexa Fluor 680, also significantly increased upon EHEC-L treatment compared to control conditions (Table 2).

**Table 2:** EHEC-L stimulates transcytosis of macropinocytic cargo.

**Experimental conditions**	**Transcytosis time**	**Cargo fluorescence intensity (A.U.)**	**n**	**p value**
		***Stx1***		
Control	4 h	2.7 ± 0.4	12	
	8 h	3.9 ± 0.5	12	
EHEC-L	4 h	8.8 ± 1.9*	12	0.02
	8 h	14.7 ± 2.8*	12	0.0001
EHEC-L + cytD	4 h	1.9 ± 0.6	6	NS
	8 h	2.2 ± 0.5	6	NS
		***HRP***		
Control	4 h	1.6 ± 0.6	6	
EHEC-L	4 h	3.8 ± 1.0*	6	0.04
		***40kDadextran***		
Control	4 h	28.5 ± 3.4	5	
EHEC-L	4 h	87.0 ± 16.9*	6	0.03

Fluorescence intensity (A.U.) of transcytosed Stx1, HRP or dextran normalized to background fluorescence intensity in control and experimental conditions; * - significant versus corresponding controls; NS – not significant versus corresponding controls; n - number of monolayers.

Additionally and in contrast to intact EHEC [20,44], the EHEC-L did not decrease the TER of T84 monolayers at any experimental time point (up to 24 h) and the TER of 1 mg/mL EHEC-L-treated monolayer (1,986 ± 205 Ω·cm^2^) was indistinguishable from the control monolayers (2,058 ± 167 Ω·cm^2^, n=48 monolayers per condition, p < 0.001), further suggesting that transcytosis of the cargo occurs via a transcellular and not paracellular pathway. Also, TEM indicates that EHEC-L-induced apical blebbing is not the hallmark of massive cell death.

Taken together, these data indicate that bacterial factors, through actin remodeling, induce a novel pathway for transepithelial delivery of Stx1 and Stx2 and possibly other antigens from the apical to basolateral side of the intestinal epithelium.

### EspP present in EHEC-L is sufficient to stimulate toxin MPC *in vitro* and *in vivo*

**Figure 8:**
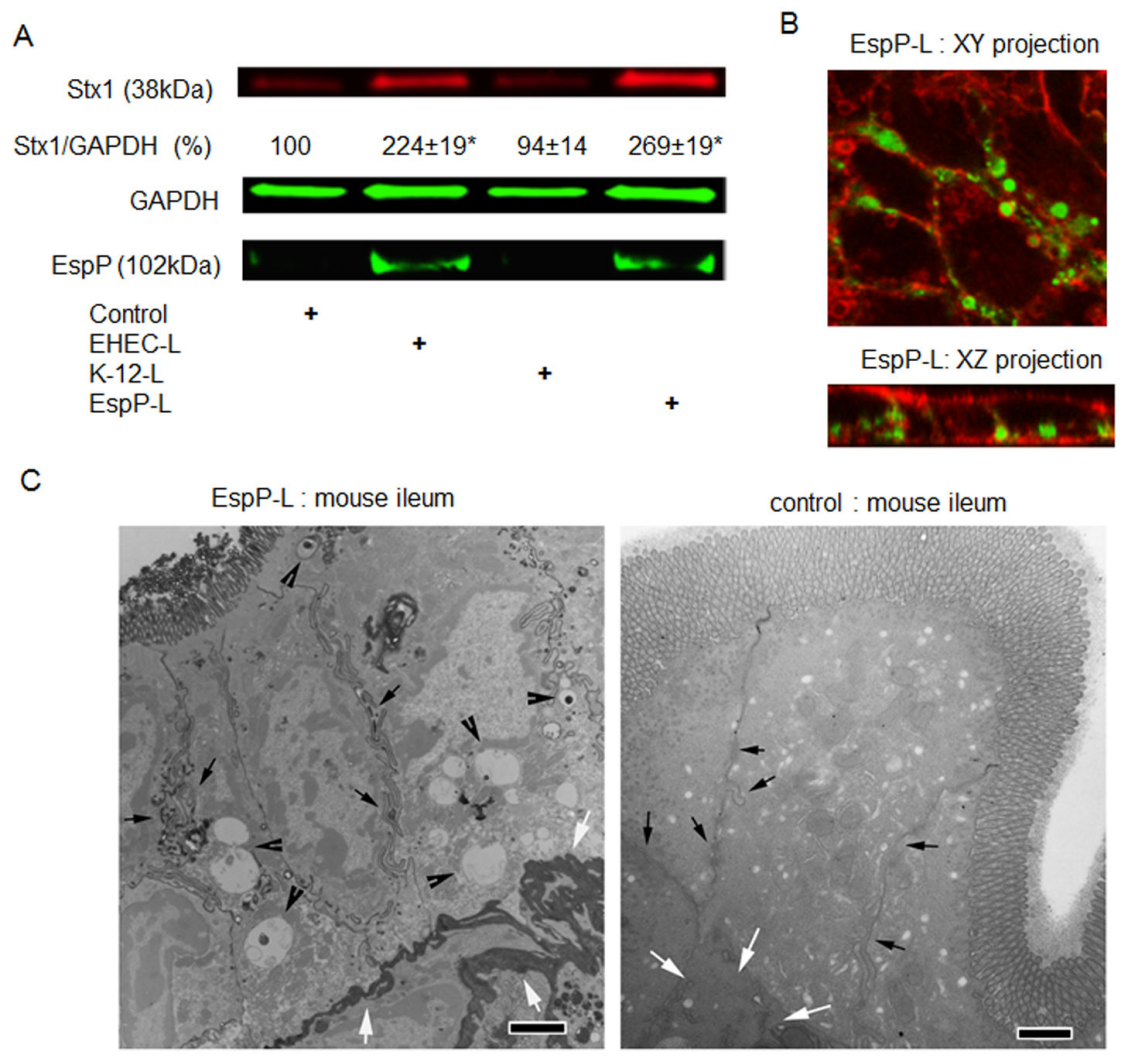
**Serine protease EspP is sufficient to stimulate Stx1 MPC in T84 cells.** (A) Representative IB and quantitative representations of IB data show that EspP expression by bacteria is sufficient to significantly increase Stx1 uptake in T84 cells compared to untreated cells or cells treated with lysates from K-12 bacteria that do not express EspP (n ≥ 3 monolayers per experimental condition; * - significant compared to the control (p < 0.05)). (B) Representative confocal optical sections through T84 cells incubated for 4 h in the presence of EspP-L (1 mg/mL) and B-subunit of Stx (Stx1B; 0.5 µg/mL) show that EspP reorganized actin in T84 cells and triggered the formation of actin coated macropinosomes that filled with Stx1B. F-actin - red by phalloidin-AlexaFluor568; Stx1B - green by AlexaFluor 488. (C) Representative TEM images of mouse ileal tissue treated from the luminal side with either a mixture of EspP-L and 2 mg/mL HRP or with mixture of K-12-L and 2 mg/mL HRP (control), bar -2 µm. EspP-L caused the formation of macropinosomes (black arrowheads) often containing HRP (black vesicles inside the macropinosomes). The macropinosomes are completely absent from control tissue. Importantly, EspP-L treatment caused the HRP accumulation in *lamina propria* (white arrows), which indicates the HRP transepithelial delivery. In contrast, HRP was absent from *lamina propria* (white arrows) in control tissue. Macropinosomes were often concentrated close to the lateral membranes (small black arrows) in ileal tissue, similar to observations in T84 cells, bars -2 µm.

It has been previously reported [42,45] that serine protease autotransporters of *Enterobacteriaceae* (SPATEs), namely Pet (plasmid-encoded toxin) from EAEC and EspC (*E. coli* secreted protein C) from enteropathogenic *E. coli* (EPEC) strains, possess a consensus serine protease motif that causes actin remodeling in IEC. Many EHEC strains including EDL933 also express a SPATE family member termed EspP (*E. coli* secreted protein P). Thus, we tested a hypothesis that EspP might be responsible for stimulation of MPC in T84 cells. We took advantage of a previously reported laboratory strain *E. coli* K-12 transformed with the *espP* gene [46]. T84 cells were treated with lysates (0.3 mg/mL) prepared from either K-12-EspP strain (EspP-L), parental K-12 strain (K-12-L) that naturally lacks EspP, or EHEC-L, each in the presence of Stx1 for 4 h. The amount of endocytosed Stx1 was measured in total cell lysates (Figure 8A). EspP-L was sufficient to stimulate Stx1 uptake compared to K-12-L or control cells not exposed to bacterial lysates. Moreover, the amount of Stx1 internalized by EspP-L-treated T84 cells was similar to cells treated with EHEC-L. Surprisingly, EspP was also internalized by T84 cells and the amount of endocytosed Stx1 correlated with the amount of EspP in T84 cell lysates. This EspP-induced increase in Stx1 uptake was accompanied by significant actin remodeling (Figure 8B) with toxin residing inside actin-coated macropinosomes, similar to what we have detected in T84 cells treated either with EHEC [20], EHEC-L, or EAEC-L. These data indicate that EHEC-expressed serine protease EspP is capable of stimulating Stx1 uptake in IEC through actin remodeling and formation of macropinosomes in vitro.

Consequently, we tested whether EspP-L stimulates MPC and possibly transcytosis of macropinocytic cargo *in vivo*. Because multiphoton microscopy resolution (Figure 5C) was not sufficient to resolve the intracellular vesicles inside the mouse enterocytes (due in part to tissue autofluorescence, light scattering, and smaller cell size in tissue compared to cultured T84 cells), we applied TEM to detect EspP-induced changes in tissue. For these experiments, two 1 cm loops were created in each mouse (n = 2 animals). Loops were injected with 2 mg/mL HRP and either 1 mg/mL K-12-L (control) or 1 mg/mL EspP-L (experimental). To detect possible HRP transcytosis into the *lamina propria*, we extended the experimental time to 6 hours, after which mice were sacrificed and tissue fixed and prepared to detect HRP by TEM.

EspP induced the appearance of macropinosomes which varied in size and shape (Figure 8C). A number of macropinosomes carried HRP inside. HRP-bearing vesicles were often concentrated near basolateral membranes, a potential site of HRP transcytosis. Importantly, HRP was readily detectable in the submucosa, demonstrating that EspP-L treatment leads to transepithelial trafficking of luminal macropinocytic cargo and release of cargo (in this case HRP) into the *lamina propria* in mouse ileum. All mentioned observations, including macropinosomes (empty or HRP-bearing) and HRP transcytosis were not detected in control samples. EspP treatment also damaged the brush border of mouse enterocytes compared to the control mouse enterocytes (Figure 8C). We conclude that EspP is sufficient to trigger macropinocytosis of high molecular weight cargo *in vivo*, which results in the transepithelial delivery of macropinocytosed material from the mucosal to serosal side.

In conclusion, EHEC-induced actin remodeling that is necessary for Stx MPC and transcytosis does not require active EspA-mediated type 3 secretion or intimin-mediated attachment, and is different from mechanisms of actin remodeling involved in pedestal formation. EHEC soluble factor(s), particularly serine protease EspP, is sufficient to stimulate Stx MPC and transcellular transcytosis *in vitro* and *in vivo*. Importantly, soluble factor(s) from another deadly enteric pathogen, EAEC H104:O4, is also able to stimulate a similar pathway leading to significant increase in Stx uptake.

## Discussion

The 2011 outbreak of STEC diseases started in Germany, spread through 16 countries and underscored the public health importance of this type of foodborne pathogen [12,13]. Several features make STEC particularly worrisome. New extremely virulent STEC strains different from classical EHEC O157:H7 are evolving [1–4,6,7]. The number of EHEC-related outbreaks has increased markedly in recent years worldwide along with an increase in economic burden and deaths. Once they are established, there are no effective treatments for intestinal or systemic STEC illnesses [4,6]. Antibiotics given for STEC-related diarrhea, particularly those that target bacterial DNA, increase the risk of developing HUS [4,47]. Soluble multivalent Gb3 receptor-based Stx1 and Stx2 binding agents [6,16] did not succeed as an anti-toxin treatment when administered in the intestine. Better characterization of the molecular mechanisms of Stx1 and Stx2 uptake and transcytosis by human enterocytes, the gateway to systemic dissemination of these toxins, could identify targets for novel therapeutic approaches for STEC diseases.

The present study provides insights into the molecular mechanism of Stx1 and Stx2 uptake by human enterocytes in the absence of Gb3 receptors and examines transcytosis across the intestinal epithelial barrier at the earliest stage of EHEC infection, ahead of significant ischemia and inflammation. Our current data suggest that EHEC infection stimulates toxin endocytosis and transcytosis by enterocytes, initiating the actin remodeling that leads to toxin MPC. This actin rearrangement necessary for toxin MPC and transcellular transcytosis is independent of type 3 secretion and intimin attachment. Several lines of evidence indicate that formation of actin pedestals and macropinosomes occur by two distinct actin polymerization-depolymerization pathways orchestrated by EHEC that serve different goals in EHEC pathogenesis. The end point of T3SS-mediated actin remodeling is the anchoring of the bacteria to the apical surface of enterocytes. The result of MPC is a transfer of high molecular weight luminal cargo, including Shiga toxins, from the mucosal to the serosal side. Intact EHEC, while required for pedestal formation, are not necessary to stimulate MPC. Bacterial soluble factor(s) present in lysates of EHEC or EAEC is sufficient to carry out this actin rearrangement. Stimulation of MPC by bacterial lysates *in vitro* and *in vivo* results in significantly increased Stx1 and Stx2 endocytosis.

The molecular mechanisms of MPC and pedestal formation are substantially different. The molecular events involved in T3SS- and intimin-dependent EHEC attachment to IEC are well characterized [25–27]. By contrast, the mechanisms of MPC, particularly EHEC-stimulated MPC, are just emerging [20,21,24]. Comparison of effects of EHEC lysates versus intact bacteria has allowed us to begin to dissect the molecular signaling cascade necessary for toxin MPC from other aspects of bacteria-host interaction. The two processes are actin-dependent; both are inhibited by the actin-depolymerizing drug cytD [38]. However, the formation of actin pedestals requires cortactin, which is recruited by T3SS effectors to the site of bacterial attachment [37,38]. In contrast, MPC does not require cortactin and cortactin is absent from the macropinocytic blebs. The ATP-dependent motor protein NMIIA is necessary for MPC, as inhibition of NMIIA activity by drugs, shRNA [20] or MLC inhibition substantially reduces toxin MPC. Thorough analysis of cytoskeletal proteins in EHEC pedestals did not reveal NMIIA. Instead the actin binding protein tropomyosin was recruited to the sites of active actin rearrangement in these pedestals by Tir, a T3SS effector [48]. These data suggest that enteric pathogens such as EHEC that reorganize the host actin cytoskeleton during the course of infection may affect it in several ways. Some actin reorganization is pathogen-specific, allowing particular bacteria to gain the advantage in colonization, as in the case of characteristic EHEC attaching and effacing lesions. Others, including actin-dependent MPC, are less specific and likely shared among several groups of enteric pathogens. Our data showing that lysates from intimin-negative Stx-producing strain of EAEC also cause formation of apical MPC and stimulate Stx uptake by IEC strongly supports this suggestion. These data are also in good agreement with previously published observations that EAEC infection of T84 cells causes damage of microvilli, blebbing of apical membrane and the appearance of multiple large vacuoles in the cytoplasm of affected cells [42]. These morphological changes caused by intact EAEC are very similar to what we have observed in cells treated with intact EHEC, or EHEC-L, or EAEC-L, or EspP-L and represent MPC. Importantly, these MPC-induced morphological changes do not represent a massive cell death because the TER, which serves as an indicator of intestinal barrier function, does not decrease upon EHEC-L treatment and is similar to that in control monolayers not exposed to EHEC-L.

Analysis of EHEC soluble factors secreted independently of T3SS which might be involved in host actin remodeling suggested that serine protease EspP may be responsible for triggering MPC [42,45,46]. Indeed, lysates from EspP-expressing K-12, but not from the isogenic K-12 strain, are able to increase Stx1 uptake in IEC similar to EHEC-L and EAEC-L. This EspP-induced increase in toxin endocytosis was accompanied by significant actin remodeling, and toxin was carried into the cells and across the cells by the actin-coated macropinosomes. Importantly, similar serine proteases termed Pet are secreted by EAEC strains [42,45,49] and might be responsible for triggering the observed toxin macropinocytosis in H104:O4-induced disease.

MPC is not cargo-specific endocytosis, as indicated by uptake of Stx1 and Stx2 as well as HRP and dextran. These data suggest that high molecular weight bacterial products other than the toxins might successfully use this pathway to get inside the enterocytes. Importantly, data from us and others indicate that MPC might serve as a mechanism for movement of cargo from the intestinal lumen to the serosa while avoiding lysosomal or proteosomal degradation [20,50]. This newly recognized, actin-dependent transcellular transcytosis may represent an early antigen-presenting pathway in the intestine before TJ permeability is compromised by inflammation or other factors, and it may potentially be a major route for the systemic delivery of Stx1 and Stx2 at the earliest stages of infection. Thus, the identification of molecular targets to inhibit Stx MPC by IEC may prevent not only Stx-induced intestinal problems but also systemic complications from STEC.

The mechanisms of interaction of Stx1 and Stx2 with different types of IEC are likely different. It has been shown that Paneth cells in human small intestine bind Stx1, but not Stx2, via a proposed receptor-mediated pathway [51], although the role of Paneth cells in Stx-induced disease remains uncharacterized [7]. It has also been recently shown that M-cells, which specialize in transcellular transcytosis of antigens, can transcellularly transport not only the toxins, but also the intact EHEC [40]. This M-cell-mediated EHEC transcytosis is an intimin-independent process, similar to what we report here. Importantly, the authors showed that a non-pathogenic *E. coli* strain was not able to translocate through the M-cells, indicating that specific factor(s) present in both intimin-positive and negative EHEC strains, possibly serine proteases such as EspP, are responsible for bacteria and toxin transcytosis. However both Paneth and M-cells represent rather small sub-groups of IEC and are restricted to the small intestine. Our data indicate that factor(s) present in EHEC-L, particularly serine protease EspP, give the enterocytes, the most abundant intestinal epithelial cell type, a M-cell-like functional phenotype with regard to toxin uptake and transcellular transcytosis. The presence of Stx1 and Stx2 in enterocytes and in the *lamina propria* in both small intestine and colon in EHEC-infected patients [21] indicate that spread and uptake of Stx is not confined to the follicle-associated epithelium, a possible major site of EHEC colonization in humans [51]. Thus, secretion of MPC-induced factor(s) by EHEC significantly increases the ability of luminal toxin to interact with enterocytes far away from the attached bacteria. This may also explain why an infection with a few EHEC organisms is sufficient to cause a severe disease. Additionally, possible limitation of EHEC colonization to particular areas in the intestine (follicle-associated epithelium or ileo-cecal valve [21]) plus an estimated very low infectious dose [6] in human disease may explain why EHEC are not internalized by enterocytes via MPC.

Although our data presented here shed new light on a possible mechanism of transepithelial transport of Stx and other bacterial virulent factors in case of EHEC infection, the significance of this pathway in human disease has to be evaluated in the future. High concentrations of Stx1 and Stx2 as well as possibly EspP in our mouse and cell models might not correspond to those in human disease. However, the EspP concentration in EHEC-L did not cause T84 cell cytotoxicity, and the TER, which reflects the epithelial barrier function, did not differ from untreated cells. The previous data have also suggested that toxin production might not be constitutive at all phases of infection [52]. Lack of quantitative information about the concentration of Stx1, Stx2, or other EHEC virulent factors such as EspP in different human intestinal sections and during various stages of disease significantly limits any current modeling of EHEC-induced intestinal pathologies *in vivo* and *in vitro*.

In conclusion, EHEC or EAEC soluble factor(s), particularly serine protease EspP, is sufficient to stimulate actin remodeling independent of intimin and T3SS, which leads to Shiga toxin MPC and transcytosis across the IEC. These data suggest that many intestinal pathogens known to modify the actin-rich BB of intestinal epithelium may stimulate MPC and use this pathway to deliver the soluble virulent factors across the intestinal epithelial barrier *in vivo*. When infected with Stx-encoding phage, as happened in the case of EAEC O104:H4, this bacteria-stimulated MPC leads to a severe disease.

## Materials and Methods

### Ethics Statement

#### Vertebrate animals

All vertebrate animal experiments were performed under the direction of Laboratory Animal Medicine according to experimental protocol approved by the JHU Animal Care and Use Committee, protocol # MO10M142. The Johns Hopkins University School of Medicine takes responsibility for the humane care and use of animals in their projects and complies with the NIH Principles for the Use of Animals, the Public Health Service Policy on Humane Care and Use of Laboratory Animals by Institutions. Any unnecessary pain, discomfort or injury to animals is avoided. Restraining devices are not necessary for our project. Any mice becoming moribund are euthanized. Hopkins Animal Care and Use Committee abide by recommendations of the American Veterinary Medical Association Guidelines for Euthanasia. It consists of CO_2_ inhalation followed by cervical dislocation.

### Cells, Reagents and Antibodies

Human colonic epithelial T84 cells (ATCC, Manassas, VA) were grown and maintained in culture in DMEM (Dulbecco’s modified Eagle’s medium)/Ham’s F-12 medium (1:1) supplemented with 10% fetal bovine serum, 100 units/mL penicillin and 100 µg/mL streptomycin as we previously described [15,16]. All media were obtained from Invitrogen. For immunofluorescence, electron microscopy and transcytosis experiments, cell monolayers (passages 22-40) were grown on polycarbonate inserts with 0.4 µm pore size (Costar, Cambridge, MA) for 14-18 days. Experiments were performed on confluent monolayers with transepithelial electrical resistances (TER) > 1,500 Ω·cm^2^. For biochemical experiments, cells were grown on plastic for 7–12 days. These ages were chosen to equalize (to some extent) the stage of cell polarization on plastic vs. filters, which was estimated by similarity in relative expression of the cell polarization marker, villin (villin/GAPDH) under these two different types of cell culture conditions.

Purified Stx1and Stx2 as well as a recombinant B-subunit of Stx1 (Stx1B) were prepared as previously described [21,53]. Toxins were fluorescently labeled using Alexa Fluor protein labeling kit according to the manufacturer protocol (Invitrogen). Alexa Fluor dyes with different excitation wavelengths were used to generate the panel of toxins with different excitation properties, including Stx1-680 (Stx1 conjugated to Alexa Fluor 680), Stx2-680, Stx2-568, Stx1-488, and Stx1B-488, as we have previously described [21]. Pirl-1 was from Chembridge Co. Antibodies (Abs) were purchased as indicated: rabbit NMIIA (Covance Inc.); rabbit MLC, rabbit p-MLC and rabbit pSrcY527 (Cell Signaling), mouse GAPDH and mouse cSrc (Sigma); mouse cortactin and rabbit p-cortactin (Abcam); rabbit p-SrcY418 (Millipore). Fluorescent secondary Abs for IF (dilution 1:100), phalloidin-AlexaFluor 488 or 568 (IF dilution 1:200), and Hoechst 33342, were from Invitrogen. Fluorescent secondary Abs for IB (dilution 1:10,000) were from Rockland. All other reagents were from Sigma.

### Bacterial Strains and Lysates

EHEC strain EDL933 or O157:H7 modified to be Stx-negative were used for T84 cell infection as well as for preparation of bacterial lysates. Additionally, T84 cells were infected with EDL933 T3SS deletion mutant of *E. coli* secreted protein A (Δ*espA*) which was constructed by in-frame deletion [50,51] as described in Supporting Information, or with the O157:H7 truncation mutant of a major EHEC adhesin intimin (Δintimin) which was constructed as described [28].

Bacterial lysates from either EHEC EDL933 strain (EHEC-L), EAEC H104:O4 strain (EAEC-L), non-pathogenic laboratory strain *E. coli* K-12 (K-12-L), or strain K-12 carrying a plasmid expressing EspP [46] were prepared as previously described [54]. Briefly, each strain was grown in LB broth for 12 hours, at which time the bacteria entered stationary phase. The culture was centrifuged at 3000 x g. The bacterial pellet was washed twice with PBS then centrifuged at 3000 x g, resuspended in PBS, then lysed by sonication for 5 x 30 pulses (30% amplitude). The resulting lysate was centrifuged at 14,000 x g for 30 min and then filtered through a 0.22 µm filter.

### Infection of T84 cells by bacteria and treatment of T84 cells with bacterial lysates and pharmacologic agents

Following a published protocol [20], we inoculated T84 cells apically with either EDL933, or O157:H7, or Δ*espA*, or Δintimin strain in concentration ~10^4^ EHEC/mL and incubated them at 37 °C in 5% CO_2_ for 4 h. We had previously shown that such infection conditions do not cause significant increase in T84 cells death compared to uninfected cells [20]. Alternatively, T84 cells were apically treated with increasing concentrations of bacterial lysates EHEC-L, EAEC-L or K-12-L for 4 h at 37 °C in 5% CO_2_. Alexa Fluor 680 labeled Stx1 or Stx2 was added apically (0.3 µg/mL) at the time of treatment, as were the inhibitors cytD (0.5 µM), pirl-1 (0.5 µM), or blebbistatin (50 µM). After 4 h, the cells were washed three times with cold PBS and fixed for immunofluorescence, or lysed in RIPA buffer (1% Triton X-100, 0.5% deoxycholic acid, 0.1% SDS, 50 mM Tris HCl pH 7.4, 150 mM NaCl) containing 0.5 mM Na _3_VO_4_ and protease inhibitor cocktail (1:1000, Sigma P8340) and centrifuged at 20,000 x g at 4^°^C for 15 minutes for immunoblotting.

### Mouse intestinal loop model of EHEC-induced MPC

Mouse ileal loops were performed as described [40,41]. In general, C57BL/6 male mice were starved overnight prior to the assay. The mice were anesthetized with isofluorane and their small intestine exteriorized through a midline incision. Ligated intestinal loops that were approximately 5 cm in length were formed in the distal ileum, approximately 2 cm from the caecum. Loops were injected with 0.2 mL solution containing either 2.5 µg/mL Stx1 or a mixture of Stx1 and 1 mg/mL K-12-L (both represent the control loops), or a mixture of Stx1 and 1 mg/ml EHEC-L (experimental loop). Several pharmacological inhibitors, as listed in the results, were also added to the solution: pirl-1 (200 µM), cytD (5 µM), and blebbistatin (500 µM). After 4 h of incubation, the mice were sacrificed and the loops were removed for further biochemical experiments or immunofluorescence microscopy. For immunoblotting of mouse intestinal tissue, the excised loops were washed three times with saline then frozen at -80 °C. After addition of buffer 1 (B1, pH 7.1; 300 mM mannitol, 12 mM Tris HCl, 5 mM EGTA, 10 mM Na _3_VO_4_, 5 mM β-glycerol phosphate, 5 mM Φ-alanine, protease inhibitor cocktail at 1:1000), the tissue was homogenized on ice using a Polytron homogenizer (Brinkman Instruments, Delran, NJ) to collect the IEC. A solution of 1% Triton X-100 was added to the lysate, then rotated end-over-end for 1 h at 4 °C. The total lysate was collected after centrifugation for 10 min at 13,000 x g.

### Measurements of Stx1 and Stx2 uptake in T84 cells and mouse enterocytes

Total T84 cell lysates or total lysates prepared from mouse IEC were separated by SDS-PAGE and transferred to nitrocellulose membranes. The relative fluorescence intensity of the Stx1-680 or Stx2-680 band, which corresponds to the endocytosed toxin, was measured using a LiCor infrared imaging scanner and normalized to the fluorescence intensity of GAPDH obtained by immunoblotting, as we have previously described [20,21].

### Measurements of Stx1 transcytosis across T84 monolayers

Cells grown on polycarbonate inserts were incubated with 0.5 µg/mL Stx1-680 in the absence (basal transcytosis) or presence of 0.3 mg/mL EHEC-L (stimulated transcytosis) for the times indicated in the figure legends. At the end of the incubation, inserts were removed, 100 µL samples of media from the lower chamber containing transcytosed toxin were collected, and the relative fluorescence intensity of Stx1 was measured in triplicate using a fluorescence plate reader as we previously described [20,21]. Stx1 fluorescence intensity in conditioned media was normalized to fluorescence intensity of conditioned media from cells exposed neither to EHEC-L nor to Stx1-680. Similar experiments where done to measure the transcytosis of HRP-AlexaFluor680 (2 mg/mL) or 40 kDa dextran-AlexaFluor680 (1 mg/mL).

### Immunofluorescence

For cell immunofluorescence experiments, confluent T84 monolayers grown on filters were fixed with 3% formaldehyde in PBS for 10 minutes, washed extensively in PBS, permeabilized with 0.1% saponin, and blocked with 2% BSA and 15% FBS for 30 minutes, then incubated with primary antibodies at 4^°^C overnight. After extensive washing the cells were incubated for an additional 1 h at room temperature with fluorescently-labeled secondary antibodies, phalloidin for F-actin and Hoechst for nuclear staining, washed again, immersed in gel mount and mounted on glass slides. Fluorescence confocal imaging of cells was performed using a Zeiss 510 LSM system. Eight or twelve bit fluorescence images of confocal optical 0.4 µm sections were collected for further qualitative and quantitative analysis using MetaMorph software.

### Analysis of Stx1B uptake in mouse intestinal tissue

The transgenic mice ubiquitously expressing the N-terminal MARCKS membrane-targeted peptide fused with the fluorescent protein td-Tomato (Jackson Laboratories) [55,56] were used to determine the distribution and relative amount of Stx1 in mouse enterocytes. An approximately 1 cm piece of tissue obtained from mouse distal small intestinal loop treated for 4 h either with 0.5 µg/mL Stx1-488 alone (controls) or with a mixture of Stx1 and 1 mg/mL EHEC-L (experimental conditions) was rinsed extensively with cold PBS to remove the luminal content, opened lengthwise, glued from the serosal side to the bottom of a Petri dish and fixed with 3% formaldehyde in PBS overnight. After washing in PBS, the tissue samples from both control and EHEC-L-treated animals were subjected to fluorescence imaging using upright multiphoton microscope and 780 nm excitation source (FluoView1000, Olympus). Using an RGB emission filter set, the Stx1-488 was detected in the green channel and the td-Tomato-plasma membrane fluorescence was detected in the red channel. A piece of intestinal tissue from each mouse not exposed to toxin was used to determine the tissue autofluorescence in the green channel. Twelve bit fluorescence images of 2 µm confocal optical sections starting for the tip of intestinal villi were collected. The relative amount and distribution of Stx1 inside the mouse enterocytes in control condition and after EHEC-L treatment was quantified using MetaMorph image analysis software. Similar experiments were done using 70 kDa Dextran-Alexa Fluor 488, a fluid phase endocytosis marker with molecular weight close to that of Stx.

### Transmission electron microscopy (TEM)

For TEM, the filter-grown T84 cells or mouse ileal loops were fixed for 2 h in solution containing 2% glutaraldehyde, 2% PFA, 0.1 M Na-cacodylate, 3 mM CaCl_2_, pH 7.4 at room temperature. Samples were stored overnight in solution containing 0.1M Na-cacodylate and 3% sucrose. To detect HRP, the DAB reaction was performed for 5 min using DAB-ATA mixture in 1 M Tris buffer, pH 7.4. Then samples were incubated in 1% H_2_O_2_ for 1 h. Following 3 X 5 min buffer rinse, samples were post-fixed in 2% osmium tetroxide in 0.1 M Na cacodylate for 1 h on ice in the dark. After a brief rinse in distilled water, tissue samples were placed in 2% uranyl acetate for 1 h at room temperature in the dark. Following en-bloc staining, tissue samples were dehydrated through a graded series of ethanol to 100%, transferred through propylene oxide, embedded in Eponate 12 (Pella) and cured at 60 °C for two days. Sections were cut on a Riechert Ultracut E with a Diatome Diamond knife. Sections of 80 nm were collected on formvar coated 1 x 2 mm copper slot grids and stained with uranyl acetate followed by lead citrate. Grids were viewed on a Hitachi 7600 TEM operating at 80 kV and digital images captured with an AMT 1 K x 1 K CCD camera.

### Statistical analysis

Values are presented as mean ± SEM and the number (n) of independent preparations. Statistical significance was determined using Student’s unpaired t-test and p-value < 0.05 was considered significant.

## Supporting Information

Figure S1 Distribution of actin filaments in control T84 cells not exposed to EHEC-L. Representative singe projection from 3D reconstruction shows the F-actin distribution in grown on filter T84 cells. F-actin fibers connect lateral and basal membranes with actin perijunctional rings and do not demonstrate the vesicular structures. F-actin – green by phalloidin - Alexa Fluor 488.
(TIF)

Figure S2 Control T84 cells do not have macropinosomes. Representative TEM image of grown on filter T84 cells and treated apically for 4hours with 1mg/ml HRP shows the presence of intact microvilli at the apical surface of each cells while the large irregular vesicles that represent macropinosomes are absent from control cells. Scale bar -2 µm.
(TIF)

Figure S3 EHEC-infected T84 cells have macropinosomes similar to these induced by EHEC-L. Representative TEM image of grown on filter T84 cells and treated apically for 4 hours with EHEC and 1mg/ml HRP shows the large numbers of macropinosomes filled with HRP. Scale bar -2 µm.
(TIF)
