# Supplementary figures and images for: Serine Protease EspP from Enterohemorrhagic Escherichia Coli Is Sufficient to Induce Shiga Toxin Macropinocytosis in Intestinal Epithelium
Source: PLoS One. 2013 Jul 18;8(7):e69196. doi: 10.1371/journal.pone.0069196 (PMC3715455; doi:10.1371/journal.pone.0069196)

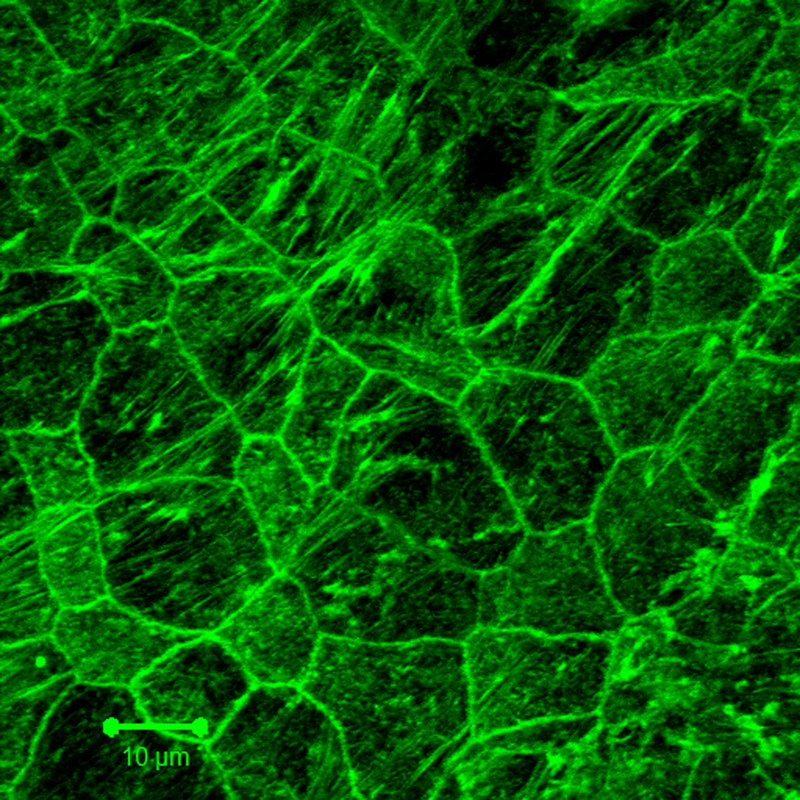

Supplement: Figure S1 — Representative singe projection from 3D reconstruction shows the F-actin distribution in grown on filter T84 cells. F-actin fibers connect lateral and basal membranes with actin perijunctional rings and do not demonstrate the vesicular structures. F-actin – green by phalloidin - Alexa Fluor 488. (TIF) [file pone.0069196.s001.tif]

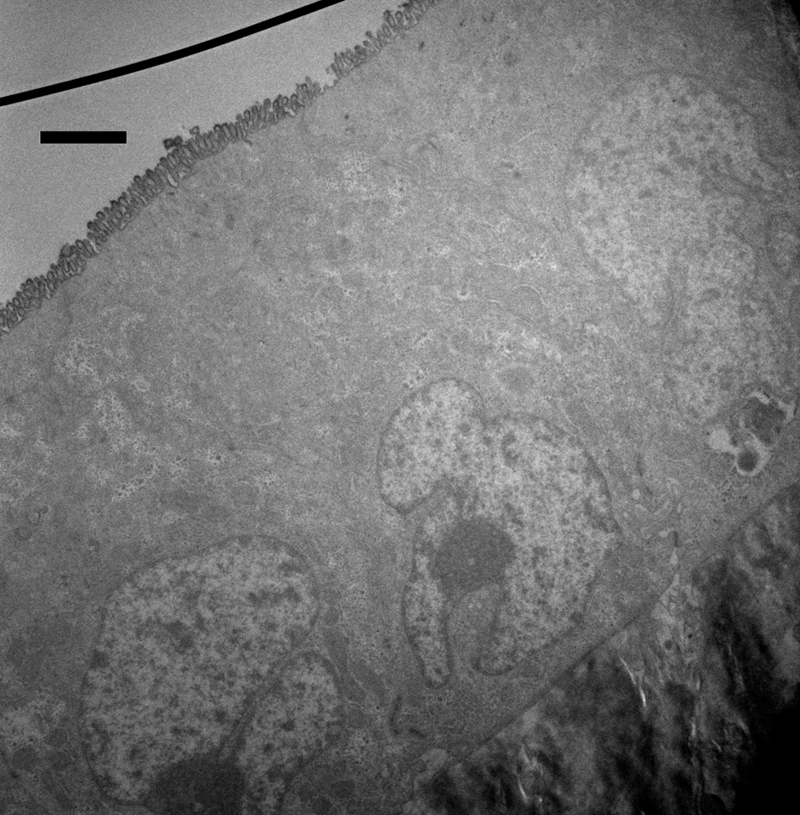

Supplement: Figure S2 — Representative TEM image of grown on filter T84 cells and treated apically for 4hours with 1mg/ml HRP shows the presence of intact microvilli at the apical surface of each cells while the large irregular vesicles that represent macropinosomes are absent from control cells. Scale bar -2 µm. (TIF) [file pone.0069196.s002.tif]

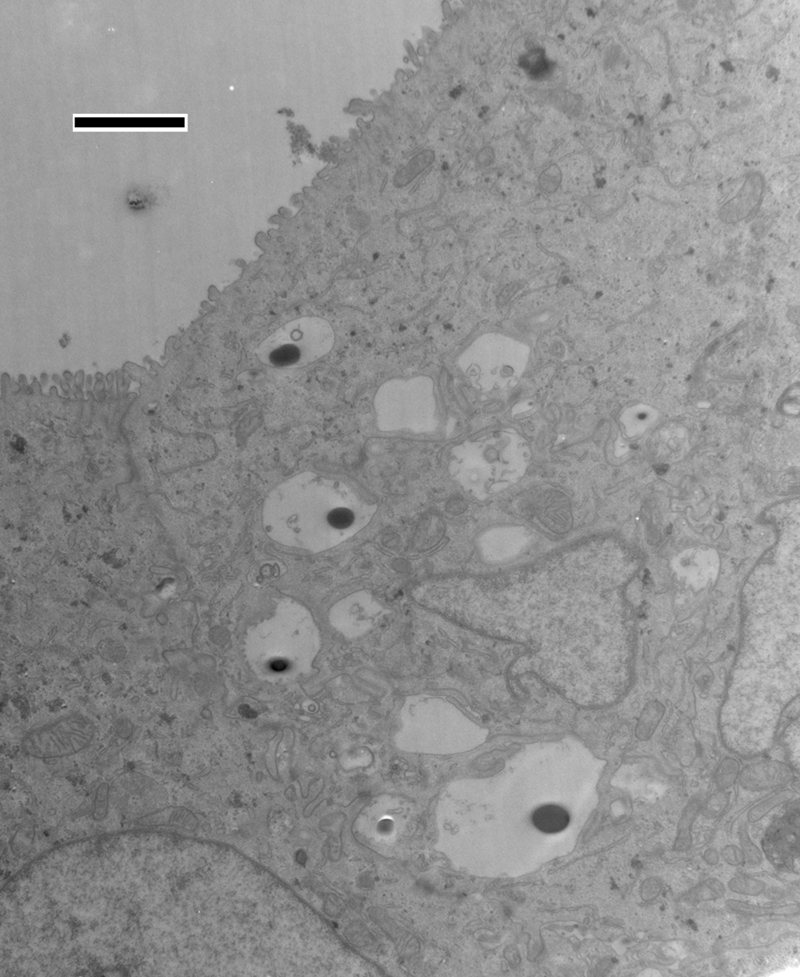

Supplement: Figure S3 — Representative TEM image of grown on filter T84 cells and treated apically for 4 hours with EHEC and 1mg/ml HRP shows the large numbers of macropinosomes filled with HRP. Scale bar -2 µm. (TIF) [file pone.0069196.s003.tif]
